# Biocomposite Coatings Doped with Magnesium and Zinc Ions in Chitosan Matrix for Antimicrobial Applications

**DOI:** 10.3390/ma16124412

**Published:** 2023-06-15

**Authors:** Daniela Predoi, Carmen Steluta Ciobanu, Simona Liliana Iconaru, Steinar Raaen, Krzysztof Rokosz

**Affiliations:** 1 National Institute of Materials Physics, Atomistilor Street, No. 405A, 077125 Magurele, Romania; ciobanucs@gmail.com (C.S.C.); simonaiconaru@gmail.com (S.L.I.); 2 Department of Physics, Norwegian University of Science and Technology (NTNU), Realfagbygget E3-124 Høgskoleringen 5, NO 7491 Trondheim, Norway; steinar.raaen@ntnu.no; 3 Faculty of Electronics and Computer Science, Koszalin University of Technology, Śniadeckich 2, PL 75-453 Koszalin, Poland

**Keywords:** biocomposite, thin film, spaceflight, chitosan, *Candida albicans*

## Abstract

Hydroxyapatite doped with magnesium and zinc in chitosan matrix biocomposites have great potential for applications in space technology, aerospace, as well as in the biomedical field, as a result of coatings with multifunctional properties that meet the increased requirements for wide applications. In this study, coatings on titanium substrates were developed using hydroxyapatite doped with magnesium and zinc ions in a chitosan matrix (MgZnHAp_Ch). Valuable information concerning the surface morphology and chemical composition of MgZnHAp_Ch composite layers were obtained from studies that performed scanning electron microscopy (SEM), X-ray photoelectron spectroscopy (XPS), energy-dispersive X-ray spectroscopy (EDS), Fourier transform infrared spectroscopy (FTIR), metallographic microscopy, and atomic force microscopy (AFM). The wettability of the novel coatings, based on magnesium and zinc-doped biocomposites in a chitosan matrix on a titanium substrate, was evaluated by performing water contact angle studies. Furthermore, the swelling properties, together with the coating’s adherence to the titanium substrate, were also analyzed. The AFM results emphasized that the composite layers exhibited the surface topography of a uniform layer, and that there were no evident cracks and fissures present on the investigated surface. Moreover, antifungal studies concerning the MgZnHAp_Ch coatings were also carried out. The data obtained from quantitative antifungal assays highlight the strong inhibitory effects of MgZnHAp_Ch against *C. albicans*. Additionally, our results underline that after 72 h of exposure, the MgZnHAp_Ch coatings display fungicidal features. Thus, the obtained results suggest that the MgZnHAp_Ch coatings possess the requisite properties that make them suitable for use in the development of new coatings with enhanced antifungal features.

## 1. Introduction

Currently, the occurrence of antimicrobial resistance has become a growing public health concern, as it could cause a significant health and economic crisis worldwide. Antimicrobial resistance occurs when microorganisms obtain the ability to become resistant to the drugs used to eradicate them [1]. One of the classes of materials used in advanced applications is composed of composite materials. This type of material is extremely attractive, even for use in aerospace applications, due to their good mechanical and physico-chemical properties [2]. 

The best-known natural composite is the bone tissue that is mainly composed of hydroxyapatite and collagen [3]. Hydroxyapatite (HAp) is one of the most used biomaterials in the medical field [4]. The unique structure of hydroxyapatite permits various types of substitutions with numerous ions (e.g., Mg, Zn, Ag, etc.) [5,6,7]. These substitutions improve both the biological and physico-chemical characteristics of hydroxyapatite [8,9,10]. The findings of the antimicrobial studies, regarding the antimicrobial activity of zinc and magnesium doped hydroxyapatite powders against Gram negative/Gram positive and fungal microbial strains (as reported by H.Alioui et al.), indicates that the metallic ions found in the powders’ compositions confer enhanced antimicrobial features to them [11]. In addition, in accordance with the studies conducted by A.N. Lipton and co-workers, good antimicrobial activity was obtained for chitosan–hydroxyapatite nanocomposite scaffolds [12]. In the study entitled “Bioactivity, in vitro corrosion behavior, and antibacterial activity of silver-zeolites doped hydroxyapatite coating on magnesium alloy” H.R. Bakhsheshi-Rad and collaborators reported that Ag-Zeo-HAp coatings exhibit enhanced antimicrobial activity against *Escherichia coli* (*E. coli*), which is caused by the TiO_2_-coated Mg alloy [13].

Chitosan is a natural polysaccharide with unique biological features such as biocompatibility, antimicrobial activity, and so on [12,14]. Titanium (Ti) is often used for biomedical applications (dentistry and orthopedics) and engineering [15]. 

Previous studies suggest that the interior of airplane cabins could be vectors for the spread of pathogens due to the high risk of contamination on the surfaces (especially seats, tables, and the lavatory), with which passengers come into direct contact [16,17,18]. In particular, the contamination of surfaces could occur due to various factors such as poor local hygiene or improper cabin cleaning procedures [16]. 

On the other hand, in accordance with World Health Organization (WHO) statements, antimicrobial resistance one of the top 10 global public health threats facing humanity due to the fact that it has the potential to affect individuals of any age, as well as the healthcare, veterinary, and agricultural industries [19,20,21,22,23,24]. The emergence of multidrug resistant infections that depend upon the use of high doses of second- and third-line drugs can harm patients due to their serious side effects. Furthermore, in some cases, these types of infections cannot be treated with conventional antimicrobial drugs, and therefore, they can pose a serious public health threat [20,21,22,23,24,25,26]. This study aimed to develop MgZnHAp_Ch thin films with antifungal properties for the first time, so that they could be used to cover the walls and interior surfaces of aircraft and even space shuttles. Taking into account the fact that *Candida albicans* is a pathogenic yeast that can be found in the air, on surfaces, and in different environmental conditions, it was chosen for testing MgZnHAp_Ch films. Fairly recent studies [27] have shown that *Candida albicans* usually parasitizes the human mouth, skin, urinary tract, and reproductive system, and it is highly adaptable to environmental conditions; its ability to adapt to environmental conditions [28] increases the risk of contamination. In addition, the studies conducted by J.A. Rosenzweig et al. [29] and T. Sugita et al. [30] showed that the risk of cross-contamination, colonization, and infection on board increases much faster in the case of the Space Station. Since the presence of *C. albicans* represents a potential risk for the health of passengers on board aircraft, as well as astronauts [31,32], a thin film of MgZnHAp_Ch can be an effective solution for protecting the interior of aircraft or space shuttles. Our results provide valuable information, and they constitute an important basis for future risk assessments that could be caused by *C. albicans*.

The *Candida albicans* (*C. albicans*) fungal strain is a pathogen that can often be found in the mouth, on the skin, or in the urinary tract of humans [33,34]. Moreover, *C. albicans*, under stressful environmental conditions (e.g., spaceflight conditions), could become virulent [29,30,33,34,35]. Therefore, in this context, the development of new advanced materials that exhibit good antimicrobial activity, and which can be used for covering cabin surfaces or metallic implants, are of great interest for the aerospace industry and for the medical community. 

In this paper, for the first time, we report results concerning the development of a new coating based on magnesium and zinc-doped hydroxyapatite in a chitosan matrix (MgZnHAp_Ch) using the dip coating method. The primary goal of this work is to improve the antimicrobial activity of titanium substrates by covering them with a biocomposite layer of MgZnHAp_Ch. Techniques such as Fourier Transform Infrared (FTIR) spectroscopy, scanning electron microscopy (SEM), energy-dispersive X-ray spectroscopy (EDS), metallographic microscopy, Atomic Force Microscopy, water contact angle, swelling, and tape-pull tests were used for the complex characterization of the MgZnHAp_Ch coatings deposited on the titanium substrate. In addition, their antifungal properties against the *Candida albicans* fungal strain were tested.

## 2. Materials and Methods

### 2.1. Materials

The precursors used for the development of MgZnHAp_Ch sample were: (NH_4_)_2_HPO_4_; Ca(NO_3_)_2_·4H_2_O; NH_4_OH; Zn(NO_3_)_6_·6H_2_O; Mg(NO_3_)_2_·6H_2_O; chitosan (low molecular weight, 75–85% deacetylated); and absolute ethanol and deionized water (DIW). All these chemical reagents were purchased from Sigma Aldrich (St. Louis, MO, USA) and used without further purification.

#### 2.1.1. Development of the MgZnHAp_Ch Biocomposites

The adapted sol-gel route used for the fabrication of the MgZnHAp_Ch sample (x_Zn_ = 0.07 and x_Mg_ = 0.05) was previously detailed in [36]. Firstly, the value of the [Ca + Mg+ Zn]/P molar ratio was 1.67. Moreover, a solution containing (NH_4_)_2_HPO_4_, Mg(NO_3_)_2_·6H_2_O, Zn(NO_3_)_6_·6H_2_O was dropped (under vigorous stirring) in a solution containing chitosan. Next, the obtained solution was dropped in a solution containing Ca(NO_3_)_2_·4H_2_O. The pH of the final solution was maintained at 11 with the aid of NH_4_OH. Afterwards, the MgZnHAp_Ch solution was mixed for 48 h at 100 °C. The MgZnHAp_Ch sols were finally added to a chitosan solution.

#### 2.1.2. Development of the MgZnHAp_Ch Composite Coatings on the Titanium Substrate

For these experiments, commercially available titanium foil (Ti, Alfa Aesar, Ward Hill, MA, USA) was used as the substrate. Next, the titanium foil was cut into pieces of 1 × 1 cm^2^. Prior to the deposition of the coatings, the Ti substrate was degreased several times using acetone in an ultrasonic bath. The deionized water was used for rinsing the substrate after each time degreasing occurred. The MgZnHAp_Ch composite coatings were developed with the aid of the dip coating method [37]. The Ti substrate was dipped for several minutes (for 5 times) into the MgZnHAp_Ch sols and drawn out with the aid of a homemade device using a lifting speed of 7 μm/min. All the dip coating procedure steps were performed at room temperature. After each dipping step, the coatings were dried at 80 °C for 30 min. Finally, the composite coatings were dried in air at 100 °C for 24 h. 

### 2.2. Methods

For the XPS measurements, an SES 2002 instrument (Scienta Omicron, Taunusstein, Germany) equipped with a monochromatic Al K(alpha) (hν = 1486.6 eV) X-ray source (Scienta Omicron, 18.7 mA, 13.02 kV, Taunusstein, Germany) was used. The recorded XPS experimental data were analyzed with the aid of Casa XPS 2.3.14 software (Shirley background type, Casa Software Ltd., Las Vegas, NV, USA) [38]. The obtained binding energy (BE) values were charge-corrected to C 1s at 284.8 eV. 

The Fourier Transform Infrared (FTIR) spectra of the MgZnHAp_Ch composite coatings were obtained using the Jasco FTIR-6600 spectrometer (Easton, MD, USA) in Attenuated Total Reflection (ATR) mode. The characteristic FTIR spectra of the MgZnHAp_Ch composite coatings were recorded between 450 and 4000 cm^−1^ with a spectral resolution of 4 cm^–1^.

For the scanning electron microscopy (SEM) and energy-dispersive X-ray spectroscopy studies, a Hitachi S4500 microscope (Hitachi, Tokyo, Japan), together with an EDAX device, were used. The quantitative elemental composition of the composite layers was also obtained using EDS. In order to obtain the 3D images of the SEM micrographs, ImageJ 1.51j8 software was used [39]. Moreover, the thickness of the composite layers was determined using transversal cross section SEM images.

Complementary information concerning the surface topography of the MgZnHAp_Ch biocomposite layers was gathered via metallographic microscopy evaluations. The samples were visualized using the 50× magnification objective of an inversed trinocular metallographic microscope, OX.2153-PLM (Euromex, Arnhem, The Netherlands). The obtained images were processed with the aid of Image J software (Image J 1.51j8) [39]. The same software was used for obtaining the 3D images of the metallographic images [39].

Information regarding the surface topography of the MgZnHAp_Ch composite layers was obtained by performing AFM studies using a NT-MDT NTEGRA Probe Nano Laboratory instrument (NT-MDT, Moscow, Russia). For the determination of the surface topography of MgZnHAp_Ch composite layers, the instrument was used in semi-contact mode, in atmospheric conditions, and at room temperature. The AFM surface images of the MgZnHAp_Ch biocomposite coatings were registered on a surface area of 15 × 15 µm^2^. The roughness parameter R_RMS_ was also calculated for the MgZnHAp_Ch composite layers, for three zones, and it was presented as the mean ± standard deviation. The obtained AFM data were processed using Gwyddion 2.59 software (Department of Nanometrology, Czech Metrology Institute, Brno, Czech Republic) [40].

For the water contact angle measurements, a contact angle goniometer (DSA30 Kruess GmbH, Germany) was used. The procedure was conducted under ambient conditions, in accordance with the conditions reported in our previous work [41]. In this paper, the values of θ (°) ± SD are reported.

For the swelling test, dried MgZnHAp_Ch composite coatings were weighted (*E_d_*). Subsequently, MgZnHAp_Ch composite coatings were immersed in DIW for 72 h. The sample was taken out at 24 h intervals, and the excess water was easily removed using filter paper. The wet samples were weighed (*E_w_*) and further immersed in the deionized water. This procedure was repeated 3 times, and the results of the swelling test are reported as the mean value ± standard deviation. The swelling (%) was determined using the following equation:

(1) $$\mathrm{Swelling}\;(\%)=\frac{E_{w}-E_{d}}{E_{d}}\times 100$$

The tape-pull test was used for the evaluation of the adherence of MgZnHAp_Ch composite coatings to the titanium substrate. The 3M Performance Flatback Tape 2525 tape was used in order to perform coating adhesion studies. The peel adhesion of the tape used in this experiment was 7.5 N/cm.

### 2.3. In Vitro Antifungal Assay

The in vitro antifungal assays were used against the reference fungal strain, *Candida albicans* ATCC 10231. The experimental conditions were previously described in [6], and the antifungal activity of the biocomposite layers was evaluated at three different time intervals (24, 48, and 72 h) during incubation with fungal suspensions. All the experiments were carried out in triplicate, and the results were presented as the mean ± standard deviation. The statistical analysis was carried out using the student t-test and analysis of variance (ANOVA). The value of statistical significance was *p* < 0.05. For the AFM qualitative observation, the MgZnHAp_Ch composite layers were taken out of the *C. albicans* ATCC 10231 culture medium after 3 intervals of incubation (24, 48, and 72 h). Then, the specimens were washed with a sterile saline solution and finally fixed with cold methanol.

## 3. Results

**Figure 1:**
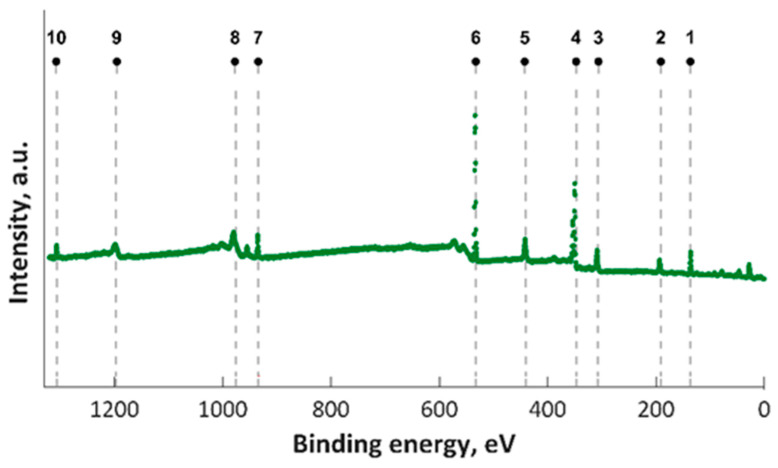
XPS survey results for MgZnHAp_Ch examined samples; 1—P 2p, 2—P 2s, 3– Mg KLL, 4—Ca 2p, 5—Ca 2s, 6—O 1s, 7—Zn 2p, 8—O KLL, 9—Ca LMM, and 10—Mg 1s.

In this study, XPS investigations into MgZnHAp_Ch composite thin film were carried out. The presence of magnesium and zinc ions on the surface was underlined in the studies. The constituent elements of the MgZnHAp_Ch layer (Ca, P, O, Mg (1s), Mg (KLL) and Zn) were observed in the general XPS spectrum (Figure 1). The signal that was recorded for C1s arose from the reference carbon with a binding energy (BE) of 289.5 eV. The XPS findings revealed that the upper layer of the MgZnHAp_Ch biocomposite coating contained zinc (Zn^2+^), magnesium (Mg^2+^), and calcium (Ca^2+^) from hydroxyapatite (HAp). The binding energy signal of Ca 2p, O 1s, and P 2p was found to be in accordance with earlier reported studies [42,43,44], and with the value for hydroxyapatite (HAp).

**Figure 2:**
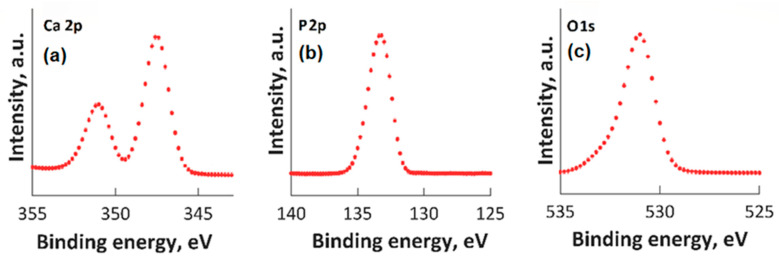
High-resolution XPS spectra for Ca 2p (**a**), P2p (**b**), O1s (**c**).

High-resolution XPS spectra for Ca 2p, O 1s, and P 2p are presented in Figure 2. In the XPS spectrum of O1s (Figure 2c), the maximum result was observed at 531.2 eV. This maximum result was assigned to Hap, and it is in accordance with prior reports [42,43,44]. In accordance with anterior studies [42,43,44], both the peak observed at 133.2 eV (for the binding energy of P 2p (Figure 2b)) and the peak associated with Ca 2p (Figure 2a) (that was identified at approximately 347.3 eV) are in accordance with anterior reported results [42,43,44]. 

**Figure 3:**
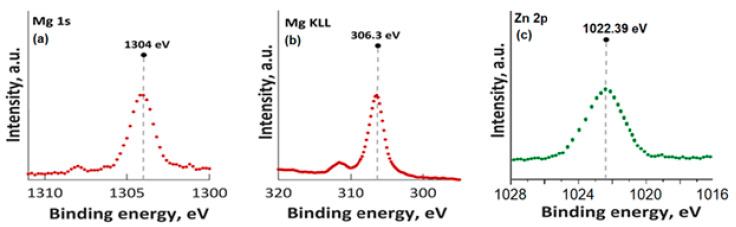
High-resolution XPS spectra for Mg 1s (**a**), Mg KLL (**b**), and Zn 2p (**c**).

In Figure 3, the high-resolution XPS spectra for Mg 1s, Mg KLL, and Zn 2p are presented. It was observed that the positions of the Mg 1s and Mg KLL peaks (Figure 3a,b) were observed at binding energies of 1302 and 304.3 eV. Regarding the XPS spectra of MgZnHApCh, the associated Zn 2p maximum value, present in Figure 3c, was found at a BE of approximately 1022.41 eV. This binding energy value, associated with the specific peak for Zn 2p, aligns well with the data that were previously presented in the literature [45]. Moreover, in accordance with the data presented by S. Feliu et al. [46], the presence of the peak associated with Zn 2p at this value suggests that following the substitution of Ca^2+^ ions, the valence of Zn was not modified.

**Figure 4:**
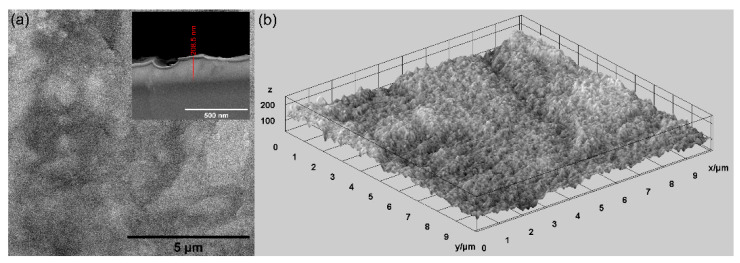
The SEM 2D SEM image and inset of cross section (**a**), and 3D (**b**) images of the MgZnHAp_Ch composite coatings’ surface.

In this study, various techniques were used for the evaluation of the surface morphology of MgZnHAp_Ch composite coatings deposited on titanium substrates. Firstly, scanning electron microscopy (SEM) was utilized for the evaluation of the surface morphology, and the results are shown in Figure 4. In Figure 4, both the 2D and 3D SEM images are shown. 

As is evident, the obtained SEM micrographs reveal the presence of a compact, relatively homogenous and continuous MgZnHAp_Ch layer. Therefore, using an adapted sol-gel method can obtain composite MgZnHAp_Ch layers with a relatively smooth surface morphology. Moreover, Figure 4 highlights the absence of fissures and/or cracks on the surface of the studied biocomposite coatings. Moreover, the thickness of the MgZnHAp_Ch composite layer was estimated using the transversal cross section SEM image, depicted as an inset in Figure 4a. The thickness of the MgZnHAp_Ch composite layer, as shown in the transversal cross section SEM image, was approximately 208.5 nm.

**Figure 5:**
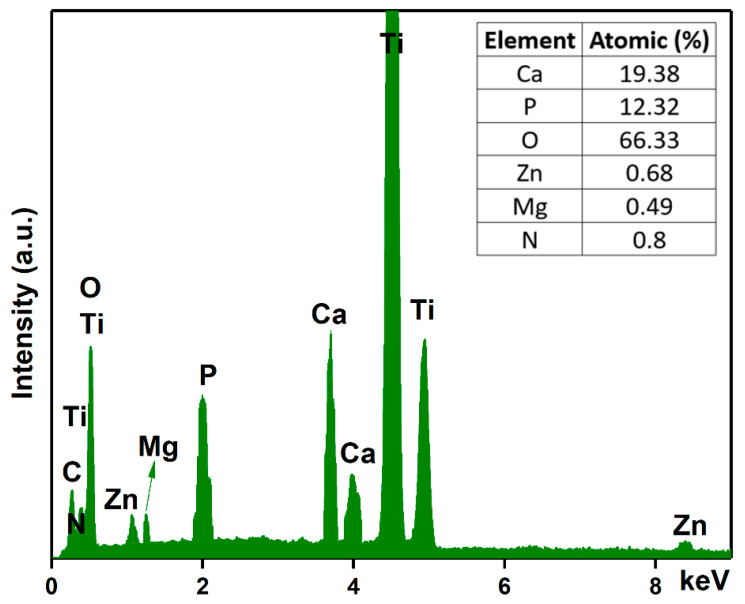
Energy-dispersive X-ray spectra and the elemental composition estimated for the MgZnHAp_Ch composite coatings.

Another objective of this work was to analyze the chemical composition of the MgZnHAp_Ch composite coatings. For this purpose, energy-dispersive X-ray spectroscopy (EDS) analyses were conducted, and the results are shown in Figure 5. The experimental results shown in Figure 5 suggest that the obtained composite coatings are pure. This is supported by the lack of supplementary lines in the EDS spectra, which is characteristic of the MgZnHAp_Ch sample. Furthermore, only the presence of Mg (Magnesium), Zn (zinc), C (Carbon), O (Oxygen), P (Phosphorus), Ca (Calcium), and N (Nitrogen) can be easily observed in the EDS spectra. These constituent elements are the major constituents of the MgZnHAp_Ch coatings’ structure. The presence of the Ti (Titanium) line in the EDS spectra is due to the substrate. Furthermore, the results of the energy dispersive X-ray spectroscopy (EDS) quantitative analysis (inset of Figure 5) confirm the presence of both zinc and magnesium ions in the samples, as well as the constituent elements of the hydroxyapatite and chitosan matrix, respectively.

**Figure 6:**
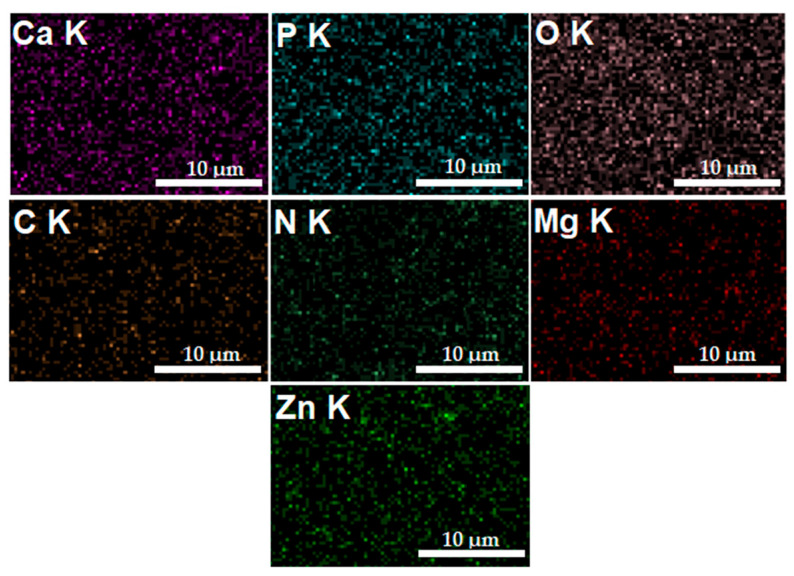
The Ca, O, P, C, N, Zn, and Mg elemental distribution maps obtained from the MgZnHAp_Ch coatings.

In Figure 6, the elemental distribution maps that are characteristic of the MgZnHAp_Ch coatings are presented. The elemental distribution maps reveal valuable information concerning the good and homogeneous distribution of Mg (Magnesium), Zn (zinc), C (Carbon), O (Oxygen), P (Phosphorus), Ca (Calcium), and N (Nitrogen) in the MgZnHAp_Ch composite coatings. Moreover, the elemental distribution maps also suggest the coatings’ purity. 

**Figure 7:**
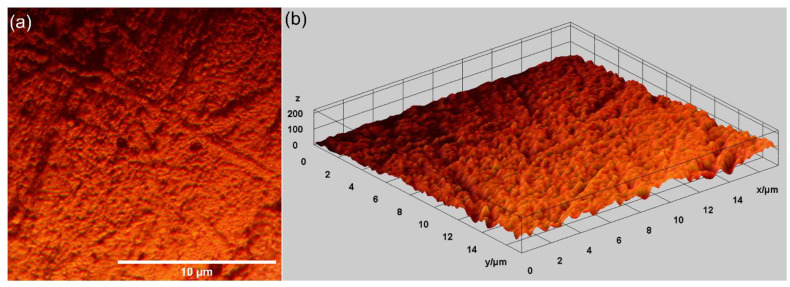
Two-dimensional images obtained from the metallographic studies of MgZnHAp_Ch composite layers (**a**) and their 3D representation (**b**).

Other relevant information concerning the morphological features of the MgZnHAp_Ch composite layer’s surface was determined using metallographic microscopy studies. The 2D metallographic image of the MgZnHAp_Ch composite layers, obtained using the 50× of the MM, as well as the 3D representation, are presented in Figure 7. 

The results of the metallographic microscopy observations underline that the surfaces of MgZnHAp_Ch composite layers do not exhibit any signs of cracks, fissures, or impurities. In addition, the 3D metallographic images of the 2D metallographic image prove that the surface of the MgZnHAp_Ch composite layers exhibit the typical appearance of a continuous and relatively uniform deposited layer. The data obtained using metallographic microscopy align well with the results provided by the SEM measurements, which show that the MgZnHAp_Ch composite layer’s surface is free of any significant surface defects, such as cracks or fissures, or other impurities, and they attest to its continuity and uniformity. 

**Figure 8:**
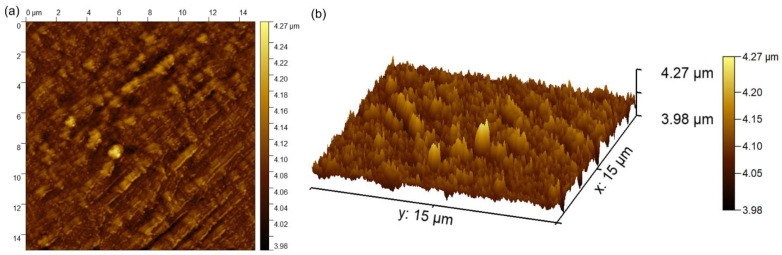
The 2D (**a**) and 3D (**b**) AFM surface topography of MgZnHAp_Ch composite layers.

Complementary information concerning the surface topography of the MgZnHAp_Ch biocomposite coatings were obtained using atomic force microscopy. The 2D image obtained from the surface topography of the MgZnHAp_Ch composite layers used AFM, and this image, as well as its 3D representation, are depicted in Figure 8a,b. 

The 2D AFM micrographs of the MgZnHAp_Ch composite layers, and their 3D representations, highlighted that the composite layers have the appearance of an almost uniform and continuous deposited coating. The AFM topographies of the MgZnHAp_Ch composite layers suggest that their surfaces do not present any significant cracks or any other type of discontinuity. The obtained AFM data enable the calculation of the root mean square roughness (R_RMS_) parameter. The value determined for the root mean square roughness, R_RMS_ = 26.49 nm ± 2.45, underlined that the surface topography of the MgZnHAp_Ch biocomposite thin films does not present any significant roughness and is relatively homogenous. These data are in accordance with the results of both SEM and MM studies, and they highlight that the MgZnHAp_Ch composite layers’ surfaces do not present any major defects, and that they are relatively homogenous without discontinuities. The surface characteristics of the used substrate have an important role in terms of both the final properties of the solid layer as well as the layer’s surface topography. Thus, the surface topography, and implicitly, the roughness of the coatings, are important parameters that can be used in order to modify the surface energy, and therefore, the hydrophobicity of the substrate; this can affect the amount of liquid remaining on the surface of the substrate, and consequently, the final thickness of the coating [47,48,49,50,51,52]. It is well-established that the topography of coatings’ surfaces uses a finite scale of roughness, regardless of the deposition method that was used [48,49]. For various applications (biomedical, tribological, etc.), the surface roughness is a key factor for determining the performance of the system [47,48,49,50,51,52]. The topography of the coatings generally depends on the various deposition process parameters, the deposition method, the materials used, and the nature of the substrate’s surface. In our case, the coatings’ surface topography patterns could be attributed to the Ti substrate’s topography. All the studies used to obtain information on the surface topography of the surface characteristics of MgZnHAp_Ch composite layers noted that MgZnHAp_Ch composite layers exhibit a pattern that can be attributed to the directionality of the Ti substrate topography. When a material is deposited on the surface of a solid substrate to create a coating, the surface characteristics of the substrate play an important role in the final properties of the solid layer. Our results align well with reported data from the literature, and they strengthen the fact that dip coating could be used to obtain uniform and homogenous composite layers [47,48,49,50,51,52]. In their study regarding the “Influence of roughness and grinding direction on the thickness and adhesion of sol-gel coatings deposited by dip-coating on AZ31 magnesium substrates. A Landau–Levich equation revision”, Fernandez-Hernan et al. [47] reported that the roughness of the substrate strongly contributed to the thickness of the sol-gel coatings deposited on their surfaces. Furthermore, they also determined that the direction of the grinding lines did not have a significant influence on the coatings’ thickness, but it had a strong influence on the morphology and surface topography of the coatings. Furthermore, in the studies conducted by Pawłowski et al. [52] regarding the “Electrophoretic Deposition and Characterization of Chitosan/Eudragit E 100 Coatings on Titanium Substrate”, they noted that the microstructure of the Ti grade 2 substrate, used for the electrophoretic deposition of chitosan/Eudragit E 100, also influenced the surface characteristics of the obtained coatings. Moreover, the more complex study conducted by Pawłowski et al. [51], regarding “Effects of Surface Pretreatment of Titanium Substrates on Properties of Electrophoretically Deposited Biopolymer Chitosan/Eudragit E 100 Coatings”, also determined that the surface of the substrate significantly influences the morphology and surface characteristics of the coatings. In addition, Hafit Khireddine et al. [50] also reported obtaining HA/FHA coatings with smooth and rough implant surfaces via pulsed electrodeposition, with roughness measurements in the range of 0.757 to 4.471 µm. 

Using the tape-pull test method, the adherence of the MgZnHAp_Ch composite coating to the titanium substrate was evaluated. Our results revealed that the obtained coatings adhere well to the substrate due to the fact that a small amount of composite material adhered to the scotch tape after it came off.

It is well known that, for a biomaterial, the hydrophilicity or hydrophobicity of a surface can dramatically influence the quality of their interaction with biological environments, and the interactions between biomedical devices with the surrounding tissue. Therefore, in this paper, the surface wettability properties of MgZnHAp_Ch composite coating surfaces (which were deposited on a Ti substrate) were assessed via water contact angle measurements; the data are summarized in Table 1.

The value obtained for the contact angle, θ = 59.76 ± 2.83°, reveals the presence of a hydrophilic surface. In accordance with the studies by K. Y. Law [53], obtaining a θ value under 90° highlights the presence of a hydrophilic surface. Therefore, our results align well with the results reported in the literature, and they may indicate that the composite coatings based on MgZnHAp_Ch can be promising candidates for biomedical use due to their hydrophilic surface features [5,41,54,55].

**Figure 9:**
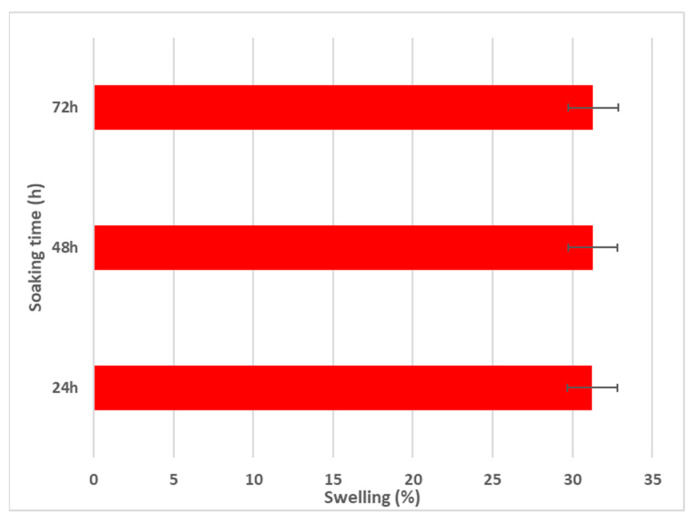
Swelling percentage of MgZnHAp_Ch composite coatings for 24, 48, and 72 h.

Using the aqueous swelling test, the water uptake capabilities of MgZnHAp_Ch composite coatings were evaluated. The swelling test data are presented in Figure 9.

As is evident in Figure 9, after 24 h of soaking in the aqueous solution, the swelling percentage reaches an almost constant value (~31%). Similar swelling ratios were obtained after 48 and 72 h of immersion. These results are in agreement with those reported by R. Ying and coworkers [56], in their work entitled “Preparation and properties of a highly dispersed nano-hydroxyapatite colloid used as a reinforcing filler for chitosan”. 

**Figure 10:**
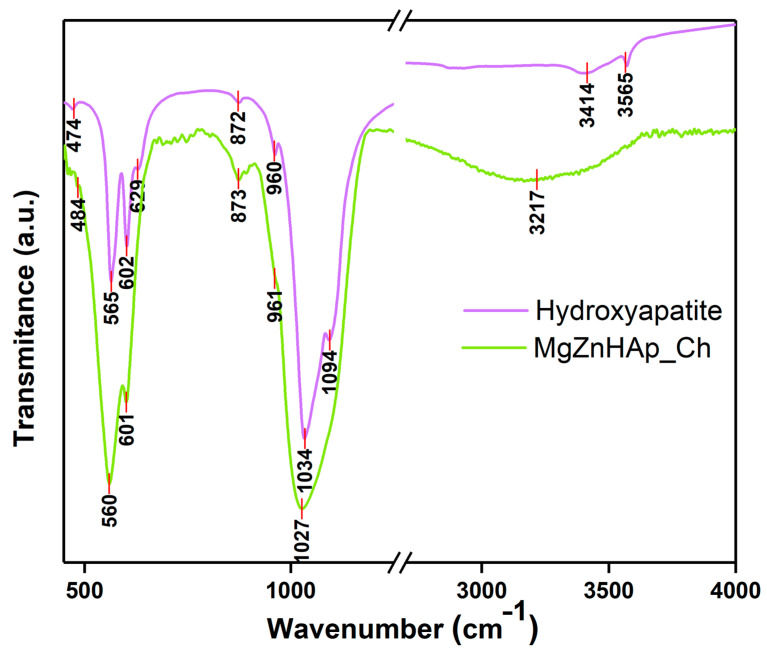
FTIR transmittance spectra of Hydroxyapatite and MgZnHAp_Ch composite coatings.

Using FTIR studies, the presence of molecular vibrations that are specific to hydroxyapatite structures in the MgZnHAp_Ch composite coatings deposited on the titanium substrate was investigated. The FTIR spectra obtained for the Hydroxyapatite and MgZnHAp_Ch samples, between 450 and 4000 cm^−1^, are shown in Figure 10.

The specific spectra of hydroxyapatite (HAp) are shown in Figure 10. As was previously reported in our studies, the maximum values found (at around 474, 565, 602, 629, 960, 1034, 1094, 3414, and 3565 cm^−1^) in the obtained spectra mainly belong to the vibrations of the phosphate/hydroxyl groups in the HAp structure [57]. As is evident from the FTIR spectra of the MgZnHAp_Ch sample, the presence of seven vibrational bands is highlighted. Five of them (at around 484, 560, 601, 961, and 1027 cm^−1^) are usually specific to the vibrations of the phosphate (PO_4_)^3−^ groups in the hydroxyapatite [58]. The first maximum value noticed at 484 cm^−1^ usually belongs to the ν_2_ of the (PO_4_)^3−^ groups. Therefore, the maximum values at 560 and 601 cm^−1^ appear due to the ν_4_ of the (PO_4_)^3−^ groups in the HAp. Furthermore, the maximum value noticed at approximately 961 cm^−1^ is characteristic of the ν_1_ vibration of the (PO_4_)^3−^ groups in HAp. Finally, the band observed at 1027 cm^−1^ correspond with the ν_3_ of the phosphate groups. On the other hand, the presence of the band noticed at approximately 3217 cm^−1^ is mainly due to the vibrations of the hydroxyl groups. As previously reported by A.G. Sánchez and collaborators [18], the vibrational bands that are specific to the chitosan structure can usually be observed in two spectral domains, namely, from 500 to 1800 cm^−1^ and from 2500 to 3500 cm^−1^ [18]. As can be seen in Figure 10, the bands of chitosan overlap with the ones in the hydroxyapatite structure. In Figure 10, it is evident that the presence of the chitosan in the sample induces a broadening spectrum of maximum values and a slight displacement of the maximum position. These FTIR characteristics are in accordance with the results previously reported in other studies [6,59]. 

**Figure 11:**
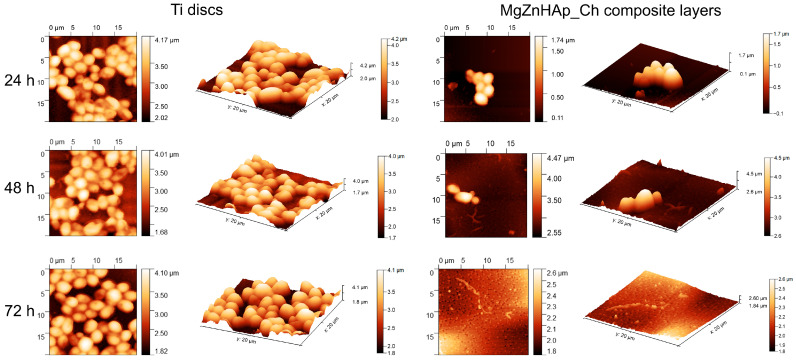
The 2D AFM surface topography of the *Candida albicans* ATCC 10231 cell development on Ti discs and MgZnHAp_Ch biocomposite coatings after being incubated over three time intervals; these results were recorded on an area of 20 × 20 μm^2^ and their 3D representation is shown.

Information regarding *C. albicans* adherence and spreading on the surface of Ti discs and MgZnHAp_Ch composite layers was gathered via AFM measurements. Thus, the AFM topographies of both the Ti discs and MgZnHAp_Ch composite layers, after being exposed to a *C. albicans* fungal suspension over three time intervals, were recorded at room temperature in semi-contact mode on a surface of 20 × 20 µm^2^. The AFM images of the Ti discs and MgZnHAp_Ch composite layers, incubated with a *C. albicans* fungal strain for 24, 48, and 72 h, are depicted in Figure 11. 

The 2D surface topography of the MgZnHAp_Ch composite layers, obtained via AFM, demonstrated that *C. albicans* fungal cell development was strongly inhibited; the level of inhibition could be related to the incubation period. Furthermore, the 2D AFM topographies also emphasized that the Ti discs did not exhibit any inhibitory activity against the *C. albicans* fungal cells. The results suggested that the Ti discs allowed the fungal cells to adhere and develop on their surfaces across all the investigated time periods. Furthermore, the 2D topography, as well as the 3D representations, suggested that Ti discs promoted the development of the *C. albicans* fungal cells on their surfaces, and provided a good adhesive surface that was suitable for the development of fungal biofilm. The 2D AFM images of the investigated samples that were previously exposed to a *C. albicans* fungal culture medium over three different time periods revealed that the morphology of the adhered cells was characteristic of *C. albicans* fungal cells; this was evident due to the ovaloid morphologies which had dimensions between 2.06 and 4.99 µm. Furthermore, the AFM results also noted that the fungal cells’ adhesion was inhibited in the early stages of incubation (in the first 24 h) by the MgZnHAp_Ch composite layers. Moreover, the AFM results noted that the inhibitory effect of the MgZnHAp_Ch composite layers, though strong during the early stage of incubation, was also related to the length of the incubation period. The AFM studies revealed that after 48 h of exposure, the fungal cells on the MgZnHAp_Ch composite layers’ surfaces were almost completely eradicated. Furthermore, the AFM investigation showed that after 72 h of exposure, the *C. albicans* fungal cells were completely eradicated from the surface of the MgZnHAp_Ch composite layers. In addition, the 2D AFM images, as well as their 3D representations, presented in Figure 11, highlighted that on the surface of the layers that were incubated in a *C. albicans* fungal suspension over three different time periods, the adhered cells were mainly isolated and there was no sign of hyphal formations that could lead to biofilm formation.

**Figure 12:**
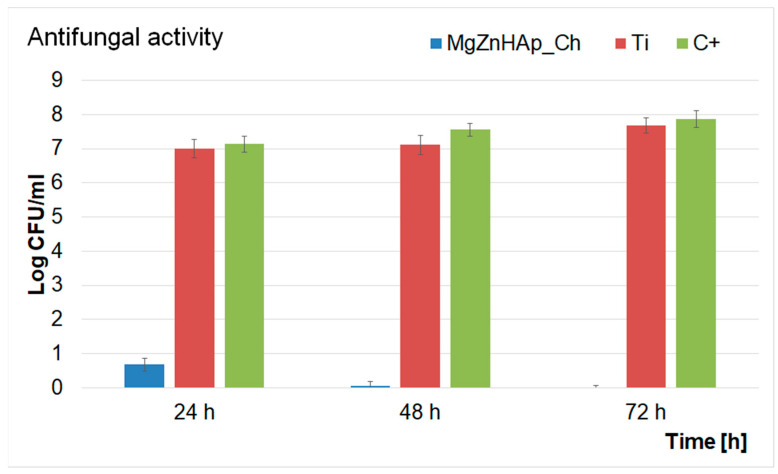
Graphical representation of the Log colony forming units (CFU)/mL of *C. albicans* as a function of length of time exposed to the Ti discs and MgZnHAp_Ch composite layers.

In addition, a quantitative test to investigate the antifungal activity of Ti discs and MgZnHAp_Ch composite layers was also performed. The results of the quantitative assays are reported in the graphical representation of the effects of the Ti discs and MgZnHAp_Ch composite layers on the adherence and development of *C. albicans* ATCC 10231 colony-forming units (CFU), over three different time periods (24 h, 48 h, and 72 h), in Figure 12. The findings of the quantitative antifungal evaluation showed that the *C. albicans* CFU’s development was strongly inhibited by the MgZnHAp_Ch composite layers from the early stages of development in the first 24 h of incubation. Moreover, the data suggest that there was a considerable reduction in the number of fungal colonies after 24 h of incubation when compared with the control (C+). In addition, the quantitative in vitro assays also emphasized that the Ti discs help promote the proliferation and adherence of *C. albicans* fungal cells to their surfaces, as shown by the considerable increase in the number of fungal colonies developed on the surface of the Ti discs when compared with the positive control. The results obtained from the quantitative antifungal evaluation determined that the antifungal features of MgZnHAp_Ch were affected by incubation time. Indeed, the CFUs decreased rapidly during the first 24 h, then, they were completely eradicated by the end of the 72 h of incubation. The results of this study are in accordance with the qualitative information obtained from the AFM studies, and with earlier studies [31,32,60,61,62,63,64].

The data obtained via the quantitative antifungal assays emphasized that the MgZnHAp_Ch exhibited strong inhibitory effects against *C. albicans* from the early stages of the incubation period. The data also highlighted that after 48 h, the number of colonies was greatly reduced, and almost to extinction. Furthermore, the data suggest that after 72 h of exposure, the *C. albicans* cells were completely eradicated from the surface of the composite layers, thus suggesting that the MgZnHAp_Ch composite layers displayed fungicidal properties after this period of time. These results were also confirmed by the AFM studies and by previously reported studies [61,62,63,64]. This behavior could be attributed either to the gradual release of magnesium and zinc ions from the hydroxyapatite, or to chitosan matrix degradation. Although novel materials based on metallic ions have recently been investigated for their antimicrobial activity, the information regarding the mechanisms involved in the antimicrobial features of these ions is still scarce [65,66,67,68,69,70,71,72,73,74,75,76,77,78,79,80]. Over the years, various studies have reported different mechanisms that could be deemed responsible for the antimicrobial properties of materials. In addition, the antimicrobial properties of materials and coatings are usually attributed to the properties of the chemical constituents of the samples, as well as the interactions that occur between them. In the case of coatings, these properties may be attributed to the interactions that take place between the coating materials and the substrate. Additionally, it has also been reported that antimicrobial activity is also strongly dependent on variables such as the nature of the microorganisms, the surface chemistry, the size and shape of the particles, the roughness of the surface (in the case of layers), as well as the length of time exposed to the pathogen [80,81,82,83]. Thus, regarding the investigated MgZnHAp_Ch biocomposite thin films, the strong antifungal properties that were revealed by the in vitro antifungal assays may be attributed to the presence of magnesium and zinc ions, chitosan, and the interactions that may emerge in biocomposite systems due to their constituent parts and the interactions between the layers and the Ti substrate. Zinc is an indispensable mineral that regulates the body’s normal functions and important systems such as the immune system, thyroid function, and blood coagulation, as well as the normal growth and development of the body’s bone system [84]. Recently, zinc ions have been found to exhibit antimicrobial properties, and they could play an essential role in the body’s immune response. One of the most common mechanisms of action of zinc ions against microbial cells is related to their ability to disrupt the membrane of microbial cells, thus inhibiting their growth and stopping their reproductive abilities. Some of the proposed mechanisms of action of zinc ions are attributed to the fact that zinc ions have the ability to bind to the negatively charged phosphate groups of the microbial cell membrane, thus disturbing the integrity of the cell and causing the leakage of some essential cellular constituents that could lead to the cell’s death. Additionally, zinc ions could also inhibit the activity of some essential enzymes such as DNA polymerases and RNA polymerases; these are essential for the microbial cell’s survival. Another possible antimicrobial mechanism of zinc ions is their ability to activate immune cells, macrophages, and neutrophils, which could boost the body’s defenses against microbial infections. The generation of reactive oxygen species (ROS), that could damage cellular components and eventually lead to cell death was also reported as a possible antimicrobial mechanism of zinc [65,66,67,70,76,78]. Overall, the proposed antimicrobial mechanisms of zinc ions rely on a combination of both the direct and indirect effects of zinc against microbial cells and the human immune system. Furthermore, magnesium is an essential element that plays a crucial role in the basic nucleic acid chemistry of all living organisms’ cells [79]. Although magnesium is well known and studied for its role in bone health and muscle function, recent studies have also demonstrated its antimicrobial properties [61,63,79,80]. Previously reported studies revealed that magnesium-based composites exhibit strong antimicrobial activity against common pathogens such as *Staphylococcus aureus*, *Pseudomonas aeruginosa*, *Escherichia coli*, and *Candida albicans*. In these studies, the antimicrobial activity of the tested compounds was attributed to the release of magnesium ions, which inhibited microbial growth by disrupting bacterial cell membranes [61,63,79,80]. In addition, magnesium has been reported to exhibit very good antifungal properties. Studies investigating the effects of magnesium ions against the common fungal pathogen, *Candida albicans*, showed that magnesium ions inhibited fungal cell growth and promoted the formation of reactive oxygen species, which have a significant toxic effect on fungal cells [61,63,76,79,80,81,82,83,84,85,86,87,88,89]. In addition, chitosan is a natural polysaccharide found in the shells of crustaceans and in the cell walls of fungi. In recent years, chitosan has attracted a great deal of attention, and it has been intensively studied due to fact that it exhibits a broad spectrum of antimicrobial activity. The most frequently reported antimicrobial mechanisms of chitosan concern the disruption of the microbial cell membrane, inhibition of enzymatic activity, the alteration of the microbial cell’s surface, and the induction of oxidative stress. For a microorganism, the cell membrane is a critical component that is responsible for controlling the exchange of nutrients and waste products with the surrounding environment. Chitosan is a biopolymer derived from chitin and it has a positively charged structure which allows it to bind with the negatively charged components of the bacterial cell membrane, such as lipopolysaccharides and phospholipids, thus destabilizing the cell membrane; this leads to the leakage of intracellular components and eventually to the cell’s death. Furthermore, chitosan can be responsible for inhibiting the activity of various enzymes, such as lysozyme, protease, and chitinase, which are involved in cell wall synthesis and in the nutrient metabolism of the microorganism. This can eventually lead to the disruption of the microbial cell wall and the accumulation of toxic metabolites that could result in the cell’s death. Another important aspect of the proposed antimicrobial mechanism of chitosan is its ability to alter microbial cells’ surfaces by binding to their surface proteins, lipids, and other macromolecules. This could lead to the inhibition of microbial adhesion, biofilm formation, and colonization, thus eventually leading to the inhibition of microbial growth and survival [68,69,71,72,73,74,75,76,77,78]. These properties demonstrated that zinc and magnesium ions, as well as chitosan, exhibit significant antimicrobial properties against various type of bacteria and fungi. The antimicrobial properties of these separate constituents make them promising candidates for the development of novel composites that could be successfully used for various applications in the food, space, pharmaceutical, and biomedical industries.

Furthermore, the results presented in this paper note that the presence of zinc, magnesium, and chitosan in the MgZnHAp_Ch composite layers confer fungicidal properties to the layers after 72 h of exposure to microbial suspensions. These results are in accordance with previously reported studies concerning the antimicrobial activity of biocomposite materials and layers comprising hydroxyapatite, zinc, and magnesium in a chitosan matrix.

In conclusion, the results of our present work align well with up-to-date reports in the literature, and they also support the hypothesis that the antimicrobial mechanisms of the coatings may be influenced by the chemical constituents, as well as the interactions between the composite materials, and between the composite layers and titanium substrate. Additionally, even if further studies are in needed in order to corroborate the potential use of these composite layers, comprising hydroxyapatite, magnesium, zinc, and chitosan, as antifungal agents, these encouraging results could serve as an important step in the future development of novel antifungal coatings for space, pharmaceutical, and biomedical-related applications.

## 4. Conclusions

In this study, for the first time, results concerning the complex characterization of MgZnHAp_Ch composite layers, developed using the dip coating method, are reported. The results of the XPS studies reveal the presence of P, Ca, O, Mg, and Zn in the MgZnHAp_Ch composite layers. The investigation of the surface morphology features of the MgZnHAp_Ch composite layers, using metallographic microscopy, revealed that the composite layers have the appearance of a relatively uniform and continuous deposited layer, with no signs of impurities, fissures, or cracks. The AFM studies emphasized that the MgZnHAp_Ch composite layer’s surface had the appearance of a relatively uniform and homogenous layer without any significant signs of unevenness or discontinuities. The chemical composition of the MgZnHAp_Ch layer was also confirmed via the EDS results. All the obtained elemental maps suggest that the constituent elements were well distributed in the analyzed samples. The presence of molecular vibrations that are specific to hydroxyapatite structures was highlighted by the FTIR data. The results of the water contact measurements underline the presence of a hydrophilic surface in the case of the MgZnHAp_Ch coatings. Our results suggest that MgZnHAp_Ch adheres well to the titanium substrate. The results of the antifungal assay revealed that our samples possess enhanced antifungal activity compared with the titanium substrate. Therefore, although more complex studies are needed, our studies emphasized that MgZnHAp_Ch coatings can represent an effective solution for protecting the interiors of aircraft or space shuttles by creating new coatings with improved antifungal features. 

## Figures and Tables

**Table 1:** Water contact angle of MgZnHAp_Ch biocomposite coating surfaces deposited on titanium substrate.

Sample	Contact Angle θ (°)
MgZnHAp_Ch	59.76 ± 2.83

## Data Availability

Not applicable.
